# Supplementary material for: Modeling glioblastoma heterogeneity as a dynamic network of cell states
Source: Mol Syst Biol. 2021 Sep 16;17(9):e10105. doi: 10.15252/msb.202010105 (PMC8444284; doi:10.15252/msb.202010105)
Supplement: Supplementary file 5 — Source Data for Figure 3 [file MSB-17-e10105-s001.zip › Figure3A_sourcedata/GSEA_3065/nosigdb_state3.GseaPreranked.1622718426028/FELDMAN_2020_S-G2.html]

Details for gene set FELDMAN\_2020\_S-G2[GSEA]

|  || Dataset | state3 |
| Phenotype | NoPhenotypeAvailable |
| Upregulated in class | na\_neg |
| GeneSet | FELDMAN\_2020\_S-G2 |
| Enrichment Score (ES) | -0.38206005 |
| Normalized Enrichment Score (NES) | -1.565447 |
| Nominal p-value | 0.0 |
| FDR q-value | 0.032827757 |
| FWER p-Value | 0.603 |
Table: GSEA Results Summary

  

Fig 1: Enrichment plot: FELDMAN\_2020\_S-G2      
 Profile of the Running ES Score & Positions of GeneSet Members on the Rank Ordered List

  

| PROBE | GENE SYMBOL | GENE\_TITLE | RANK IN GENE LIST | RANK METRIC SCORE | RUNNING ES | CORE ENRICHMENT || 1 | SCRG1 |  |  | 3 | 0.645 | 0.0116 | No |
| 2 | CENPF |  |  | 4 | 0.619 | 0.0230 | No |
| 3 | PRKDC |  |  | 6 | 0.588 | 0.0338 | No |
| 4 | PTPRZ1 |  |  | 14 | 0.532 | 0.0428 | No |
| 5 | SYNE2 |  |  | 16 | 0.529 | 0.0524 | No |
| 6 | PLAT |  |  | 18 | 0.513 | 0.0618 | No |
| 7 | ASPM |  |  | 25 | 0.480 | 0.0700 | No |
| 8 | GPM6A |  |  | 37 | 0.460 | 0.0772 | No |
| 9 | TOP2A |  |  | 42 | 0.448 | 0.0850 | No |
| 10 | GGH |  |  | 46 | 0.439 | 0.0928 | No |
| 11 | MALAT1 |  |  | 57 | 0.412 | 0.0992 | No |
| 12 | MKI67 |  |  | 59 | 0.407 | 0.1066 | No |
| 13 | HSP90AA1 |  |  | 60 | 0.402 | 0.1140 | No |
| 14 | ITM2B |  |  | 82 | 0.361 | 0.1182 | No |
| 15 | NASP |  |  | 85 | 0.360 | 0.1246 | No |
| 16 | GPM6B |  |  | 105 | 0.343 | 0.1287 | No |
| 17 | SMC2 |  |  | 125 | 0.322 | 0.1324 | No |
| 18 | CENPE |  |  | 126 | 0.321 | 0.1383 | No |
| 19 | DNMT1 |  |  | 149 | 0.310 | 0.1415 | No |
| 20 | KPNA2 |  |  | 157 | 0.308 | 0.1463 | No |
| 21 | PRCP |  |  | 166 | 0.303 | 0.1510 | No |
| 22 | TTYH1 |  |  | 183 | 0.293 | 0.1545 | No |
| 23 | LAPTM4A |  |  | 208 | 0.278 | 0.1568 | No |
| 24 | HELLS |  |  | 218 | 0.275 | 0.1608 | No |
| 25 | SMC1A |  |  | 231 | 0.271 | 0.1644 | No |
| 26 | DCBLD2 |  |  | 243 | 0.265 | 0.1680 | No |
| 27 | ARL6IP5 |  |  | 245 | 0.265 | 0.1728 | No |
| 28 | CTGF |  |  | 263 | 0.257 | 0.1755 | No |
| 29 | SMC4 |  |  | 265 | 0.255 | 0.1801 | No |
| 30 | TMEM59 |  |  | 269 | 0.253 | 0.1844 | No |
| 31 | ANLN |  |  | 281 | 0.249 | 0.1877 | No |
| 32 | ATAD2 |  |  | 282 | 0.249 | 0.1923 | No |
| 33 | CDH2 |  |  | 283 | 0.249 | 0.1970 | No |
| 34 | NCL |  |  | 333 | 0.234 | 0.1955 | No |
| 35 | SMC3 |  |  | 341 | 0.231 | 0.1989 | No |
| 36 | SPARC |  |  | 361 | 0.228 | 0.2009 | No |
| 37 | TNFRSF12A |  |  | 380 | 0.223 | 0.2029 | No |
| 38 | KIF20B |  |  | 411 | 0.216 | 0.2033 | No |
| 39 | TMPO |  |  | 420 | 0.214 | 0.2063 | No |
| 40 | MAGED2 |  |  | 444 | 0.210 | 0.2075 | No |
| 41 | MIS18BP1 |  |  | 448 | 0.210 | 0.2110 | No |
| 42 | NDFIP1 |  |  | 464 | 0.206 | 0.2130 | No |
| 43 | ATAD5 |  |  | 470 | 0.204 | 0.2162 | No |
| 44 | RRM1 |  |  | 537 | 0.194 | 0.2119 | No |
| 45 | TPX2 |  |  | 544 | 0.193 | 0.2148 | No |
| 46 | NCAPG |  |  | 601 | 0.186 | 0.2116 | No |
| 47 | RABAC1 |  |  | 610 | 0.185 | 0.2141 | No |
| 48 | SCD5 |  |  | 619 | 0.184 | 0.2165 | No |
| 49 | AKAP12 |  |  | 640 | 0.180 | 0.2175 | No |
| 50 | KIF23 |  |  | 662 | 0.177 | 0.2182 | No |
| 51 | HSPA8 |  |  | 678 | 0.175 | 0.2197 | No |
| 52 | NUF2 |  |  | 680 | 0.174 | 0.2228 | No |
| 53 | BAMBI |  |  | 701 | 0.170 | 0.2236 | No |
| 54 | ARHGAP11A |  |  | 702 | 0.170 | 0.2267 | No |
| 55 | SHCBP1 |  |  | 769 | 0.161 | 0.2218 | No |
| 56 | RFC4 |  |  | 835 | 0.154 | 0.2170 | No |
| 57 | MAD2L1 |  |  | 846 | 0.153 | 0.2186 | No |
| 58 | GTSE1 |  |  | 852 | 0.152 | 0.2208 | No |
| 59 | SUPT16H |  |  | 886 | 0.150 | 0.2197 | No |
| 60 | CDK1 |  |  | 915 | 0.147 | 0.2191 | No |
| 61 | CLSPN |  |  | 922 | 0.146 | 0.2211 | No |
| 62 | MCM7 |  |  | 949 | 0.143 | 0.2206 | No |
| 63 | CLU |  |  | 988 | 0.139 | 0.2187 | No |
| 64 | ORC6 |  |  | 1000 | 0.138 | 0.2199 | No |
| 65 | LMNB1 |  |  | 1021 | 0.137 | 0.2201 | No |
| 66 | NDC80 |  |  | 1087 | 0.131 | 0.2148 | No |
| 67 | BRCA2 |  |  | 1122 | 0.128 | 0.2131 | No |
| 68 | CISD2 |  |  | 1128 | 0.128 | 0.2149 | No |
| 69 | RAD51AP1 |  |  | 1196 | 0.122 | 0.2092 | No |
| 70 | RTKN2 |  |  | 1285 | 0.116 | 0.2009 | No |
| 71 | SRSF7 |  |  | 1328 | 0.113 | 0.1980 | No |
| 72 | NUSAP1 |  |  | 1338 | 0.113 | 0.1990 | No |
| 73 | ESCO2 |  |  | 1373 | 0.110 | 0.1970 | No |
| 74 | ARL6IP6 |  |  | 1390 | 0.109 | 0.1972 | No |
| 75 | TMEM237 |  |  | 1453 | 0.105 | 0.1917 | No |
| 76 | PCSK1N |  |  | 1484 | 0.104 | 0.1901 | No |
| 77 | PBK |  |  | 1524 | 0.102 | 0.1874 | No |
| 78 | HIST2H2AC |  |  | 1528 | 0.101 | 0.1889 | No |
| 79 | RNF19A |  |  | 1530 | 0.101 | 0.1906 | No |
| 80 | KIFC1 |  |  | 1605 | 0.097 | 0.1837 | No |
| 81 | HAT1 |  |  | 1609 | 0.097 | 0.1851 | No |
| 82 | SRSF2 |  |  | 1697 | 0.092 | 0.1765 | No |
| 83 | AURKB |  |  | 1838 | 0.084 | 0.1614 | No |
| 84 | MCM3 |  |  | 1918 | 0.080 | 0.1535 | No |
| 85 | CNPY2 |  |  | 1924 | 0.080 | 0.1544 | No |
| 86 | PAICS |  |  | 1931 | 0.079 | 0.1552 | No |
| 87 | CEP78 |  |  | 1933 | 0.079 | 0.1565 | No |
| 88 | EXOSC8 |  |  | 1955 | 0.078 | 0.1554 | No |
| 89 | PSAP |  |  | 1988 | 0.076 | 0.1531 | No |
| 90 | MIAT |  |  | 2016 | 0.075 | 0.1513 | No |
| 91 | PLP1 |  |  | 2047 | 0.074 | 0.1491 | No |
| 92 | MXD3 |  |  | 2058 | 0.074 | 0.1492 | No |
| 93 | IGFBP3 |  |  | 2112 | 0.071 | 0.1443 | No |
| 94 | TM7SF2 |  |  | 2168 | 0.070 | 0.1390 | No |
| 95 | CDCA3 |  |  | 2232 | 0.067 | 0.1328 | No |
| 96 | DEPDC1 |  |  | 2259 | 0.066 | 0.1309 | No |
| 97 | KPNB1 |  |  | 2293 | 0.065 | 0.1282 | No |
| 98 | PARP1 |  |  | 2393 | 0.061 | 0.1176 | No |
| 99 | DIAPH3 |  |  | 2493 | 0.057 | 0.1069 | No |
| 100 | CCNA2 |  |  | 2529 | 0.056 | 0.1038 | No |
| 101 | KIF22 |  |  | 2545 | 0.055 | 0.1030 | No |
| 102 | UBE2T |  |  | 2599 | 0.054 | 0.0977 | No |
| 103 | BTG3 |  |  | 2610 | 0.053 | 0.0975 | No |
| 104 | CDC45 |  |  | 2615 | 0.053 | 0.0980 | No |
| 105 | HES4 |  |  | 2646 | 0.052 | 0.0954 | No |
| 106 | HSPD1 |  |  | 2700 | 0.050 | 0.0901 | No |
| 107 | RRM2 |  |  | 2835 | 0.046 | 0.0750 | No |
| 108 | VRK1 |  |  | 2899 | 0.044 | 0.0684 | No |
| 109 | PARP2 |  |  | 3073 | 0.039 | 0.0485 | No |
| 110 | EIF2S1 |  |  | 3099 | 0.038 | 0.0463 | No |
| 111 | DONSON |  |  | 3191 | 0.036 | 0.0362 | No |
| 112 | KIF4A |  |  | 3195 | 0.036 | 0.0365 | No |
| 113 | HJURP |  |  | 3235 | 0.035 | 0.0325 | No |
| 114 | CKAP2 |  |  | 3236 | 0.035 | 0.0331 | No |
| 115 | SRSF3 |  |  | 3283 | 0.034 | 0.0283 | No |
| 116 | IER2 |  |  | 3345 | 0.032 | 0.0216 | No |
| 117 | TOMM40 |  |  | 3348 | 0.032 | 0.0220 | No |
| 118 | RRS1 |  |  | 3401 | 0.031 | 0.0164 | No |
| 119 | FEN1 |  |  | 3406 | 0.030 | 0.0165 | No |
| 120 | PSMC3 |  |  | 3435 | 0.030 | 0.0137 | No |
| 121 | IFITM3 |  |  | 3449 | 0.029 | 0.0127 | No |
| 122 | MELK |  |  | 3462 | 0.029 | 0.0118 | No |
| 123 | IGFBP5 |  |  | 3555 | 0.027 | 0.0014 | No |
| 124 | SPC25 |  |  | 3575 | 0.026 | -0.0004 | No |
| 125 | CKAP2L |  |  | 3787 | 0.020 | -0.0250 | No |
| 126 | ZWILCH |  |  | 3814 | 0.020 | -0.0278 | No |
| 127 | PRC1 |  |  | 3878 | 0.018 | -0.0349 | No |
| 128 | BUB3 |  |  | 3884 | 0.018 | -0.0352 | No |
| 129 | PLD3 |  |  | 3894 | 0.018 | -0.0359 | No |
| 130 | SNRPA1 |  |  | 3957 | 0.016 | -0.0430 | No |
| 131 | PCNA |  |  | 3986 | 0.016 | -0.0460 | No |
| 132 | CDCA2 |  |  | 4066 | 0.013 | -0.0551 | No |
| 133 | TRIP13 |  |  | 4114 | 0.012 | -0.0605 | No |
| 134 | FBXO5 |  |  | 4119 | 0.012 | -0.0607 | No |
| 135 | HNRNPF |  |  | 4135 | 0.012 | -0.0623 | No |
| 136 | TACC3 |  |  | 4146 | 0.011 | -0.0633 | No |
| 137 | CEP55 |  |  | 4234 | 0.009 | -0.0734 | No |
| 138 | PSIP1 |  |  | 4250 | 0.008 | -0.0751 | No |
| 139 | PA2G4 |  |  | 4363 | 0.005 | -0.0883 | No |
| 140 | CENPK |  |  | 4433 | 0.003 | -0.0964 | No |
| 141 | HNRNPDL |  |  | 4438 | 0.003 | -0.0968 | No |
| 142 | SOX9 |  |  | 4503 | 0.001 | -0.1044 | No |
| 143 | LRR1 |  |  | 4533 | 0.000 | -0.1078 | No |
| 144 | PLP2 |  |  | 4543 | 0.000 | -0.1089 | No |
| 145 | DSN1 |  |  | 4646 | -0.002 | -0.1209 | No |
| 146 | MIS18A |  |  | 4728 | -0.004 | -0.1305 | No |
| 147 | BRIX1 |  |  | 4856 | -0.007 | -0.1454 | No |
| 148 | DBF4 |  |  | 4926 | -0.009 | -0.1534 | No |
| 149 | ZWINT |  |  | 4930 | -0.009 | -0.1536 | No |
| 150 | DCK |  |  | 4959 | -0.010 | -0.1567 | No |
| 151 | C19orf48 |  |  | 4994 | -0.011 | -0.1606 | No |
| 152 | ASF1B |  |  | 5007 | -0.011 | -0.1618 | No |
| 153 | DDIT4 |  |  | 5025 | -0.012 | -0.1636 | No |
| 154 | RMI2 |  |  | 5088 | -0.013 | -0.1707 | No |
| 155 | EEF2 |  |  | 5276 | -0.018 | -0.1926 | No |
| 156 | HIRIP3 |  |  | 5291 | -0.018 | -0.1939 | No |
| 157 | PLAU |  |  | 5363 | -0.020 | -0.2019 | No |
| 158 | HMGB2 |  |  | 5479 | -0.023 | -0.2152 | No |
| 159 | CENPA |  |  | 5506 | -0.024 | -0.2178 | No |
| 160 | CENPH |  |  | 5532 | -0.025 | -0.2203 | No |
| 161 | SAE1 |  |  | 5573 | -0.026 | -0.2245 | No |
| 162 | KLF6 |  |  | 5627 | -0.028 | -0.2303 | No |
| 163 | FBL |  |  | 5681 | -0.029 | -0.2361 | No |
| 164 | CENPM |  |  | 5855 | -0.034 | -0.2559 | No |
| 165 | MND1 |  |  | 5880 | -0.035 | -0.2581 | No |
| 166 | CCDC34 |  |  | 5889 | -0.035 | -0.2584 | No |
| 167 | USP1 |  |  | 5963 | -0.038 | -0.2664 | No |
| 168 | SESN3 |  |  | 5988 | -0.039 | -0.2685 | No |
| 169 | PSMG1 |  |  | 5991 | -0.039 | -0.2680 | No |
| 170 | TUBB4B |  |  | 6059 | -0.042 | -0.2752 | No |
| 171 | PSMC3IP |  |  | 6105 | -0.043 | -0.2798 | No |
| 172 | RFC5 |  |  | 6216 | -0.047 | -0.2919 | No |
| 173 | CKLF |  |  | 6376 | -0.052 | -0.3099 | No |
| 174 | GMNN |  |  | 6392 | -0.052 | -0.3107 | No |
| 175 | HIST1H1D |  |  | 6428 | -0.054 | -0.3138 | No |
| 176 | H2AFX |  |  | 6437 | -0.054 | -0.3138 | No |
| 177 | EIF4A2 |  |  | 6481 | -0.056 | -0.3178 | No |
| 178 | TRA2B |  |  | 6483 | -0.056 | -0.3169 | No |
| 179 | CDKN2D |  |  | 6491 | -0.056 | -0.3167 | No |
| 180 | TEX30 |  |  | 6513 | -0.057 | -0.3182 | No |
| 181 | HSPB11 |  |  | 6550 | -0.058 | -0.3214 | No |
| 182 | CKS2 |  |  | 6569 | -0.059 | -0.3224 | No |
| 183 | EPS8L1 |  |  | 6584 | -0.060 | -0.3229 | No |
| 184 | IL11 |  |  | 6603 | -0.061 | -0.3240 | No |
| 185 | SPDL1 |  |  | 6606 | -0.061 | -0.3231 | No |
| 186 | DERA |  |  | 6623 | -0.062 | -0.3238 | No |
| 187 | TCF19 |  |  | 6657 | -0.064 | -0.3266 | No |
| 188 | HNRNPD |  |  | 6663 | -0.064 | -0.3260 | No |
| 189 | RBM17 |  |  | 6691 | -0.065 | -0.3280 | No |
| 190 | SAC3D1 |  |  | 6692 | -0.065 | -0.3267 | No |
| 191 | RNASEH2B |  |  | 6714 | -0.067 | -0.3280 | No |
| 192 | PGP |  |  | 6718 | -0.067 | -0.3271 | No |
| 193 | CENPW |  |  | 6750 | -0.068 | -0.3295 | No |
| 194 | RNPS1 |  |  | 6814 | -0.072 | -0.3357 | No |
| 195 | RPL22L1 |  |  | 6882 | -0.075 | -0.3423 | No |
| 196 | SNRNP40 |  |  | 6907 | -0.076 | -0.3437 | No |
| 197 | HAUS1 |  |  | 6911 | -0.076 | -0.3426 | No |
| 198 | DNAJC9 |  |  | 6958 | -0.078 | -0.3466 | No |
| 199 | FOSL1 |  |  | 7031 | -0.083 | -0.3536 | No |
| 200 | CDCA4 |  |  | 7046 | -0.084 | -0.3538 | No |
| 201 | MAGOHB |  |  | 7103 | -0.088 | -0.3588 | No |
| 202 | NUDT1 |  |  | 7124 | -0.089 | -0.3595 | No |
| 203 | RBBP8 |  |  | 7128 | -0.089 | -0.3582 | No |
| 204 | TFDP1 |  |  | 7144 | -0.090 | -0.3583 | No |
| 205 | FHL2 |  |  | 7163 | -0.091 | -0.3588 | No |
| 206 | HMGB1 |  |  | 7231 | -0.096 | -0.3650 | No |
| 207 | BEX4 |  |  | 7241 | -0.096 | -0.3643 | No |
| 208 | COPS3 |  |  | 7392 | -0.108 | -0.3801 | Yes |
| 209 | CENPV |  |  | 7393 | -0.108 | -0.3781 | Yes |
| 210 | TUBB6 |  |  | 7395 | -0.108 | -0.3762 | Yes |
| 211 | NXT1 |  |  | 7422 | -0.110 | -0.3773 | Yes |
| 212 | ACYP1 |  |  | 7427 | -0.110 | -0.3757 | Yes |
| 213 | PXMP2 |  |  | 7441 | -0.112 | -0.3752 | Yes |
| 214 | SDCBP |  |  | 7454 | -0.113 | -0.3745 | Yes |
| 215 | SNRPD1 |  |  | 7463 | -0.113 | -0.3734 | Yes |
| 216 | SSRP1 |  |  | 7499 | -0.117 | -0.3754 | Yes |
| 217 | GINS2 |  |  | 7545 | -0.122 | -0.3784 | Yes |
| 218 | RAB13 |  |  | 7553 | -0.122 | -0.3770 | Yes |
| 219 | TK1 |  |  | 7568 | -0.124 | -0.3764 | Yes |
| 220 | CKS1B |  |  | 7569 | -0.124 | -0.3741 | Yes |
| 221 | SLC25A5 |  |  | 7582 | -0.126 | -0.3732 | Yes |
| 222 | POLR3K |  |  | 7592 | -0.127 | -0.3719 | Yes |
| 223 | DHFR |  |  | 7593 | -0.127 | -0.3696 | Yes |
| 224 | CMSS1 |  |  | 7600 | -0.128 | -0.3679 | Yes |
| 225 | DUT |  |  | 7608 | -0.128 | -0.3664 | Yes |
| 226 | YPEL5 |  |  | 7616 | -0.129 | -0.3648 | Yes |
| 227 | EIF5 |  |  | 7631 | -0.131 | -0.3641 | Yes |
| 228 | SNRPB |  |  | 7671 | -0.136 | -0.3662 | Yes |
| 229 | HPRT1 |  |  | 7679 | -0.137 | -0.3645 | Yes |
| 230 | EIF1AX |  |  | 7689 | -0.138 | -0.3630 | Yes |
| 231 | CACYBP |  |  | 7708 | -0.141 | -0.3626 | Yes |
| 232 | WDR34 |  |  | 7713 | -0.141 | -0.3604 | Yes |
| 233 | TMEM106C |  |  | 7726 | -0.142 | -0.3592 | Yes |
| 234 | BIRC5 |  |  | 7735 | -0.144 | -0.3575 | Yes |
| 235 | RFC2 |  |  | 7739 | -0.144 | -0.3552 | Yes |
| 236 | CENPN |  |  | 7756 | -0.147 | -0.3544 | Yes |
| 237 | DTYMK |  |  | 7757 | -0.147 | -0.3517 | Yes |
| 238 | MYBL2 |  |  | 7771 | -0.148 | -0.3505 | Yes |
| 239 | HIST1H1E |  |  | 7780 | -0.149 | -0.3487 | Yes |
| 240 | CENPU |  |  | 7781 | -0.149 | -0.3459 | Yes |
| 241 | TYMS |  |  | 7792 | -0.150 | -0.3443 | Yes |
| 242 | STMN1 |  |  | 7808 | -0.153 | -0.3433 | Yes |
| 243 | CARHSP1 |  |  | 7812 | -0.154 | -0.3408 | Yes |
| 244 | TUBB |  |  | 7813 | -0.154 | -0.3379 | Yes |
| 245 | DCTPP1 |  |  | 7845 | -0.158 | -0.3387 | Yes |
| 246 | PFDN2 |  |  | 7875 | -0.162 | -0.3391 | Yes |
| 247 | CTNNAL1 |  |  | 7886 | -0.164 | -0.3373 | Yes |
| 248 | MZT1 |  |  | 7891 | -0.164 | -0.3347 | Yes |
| 249 | HIST1H1B |  |  | 7908 | -0.168 | -0.3335 | Yes |
| 250 | HIST1H1C |  |  | 7917 | -0.170 | -0.3313 | Yes |
| 251 | HSPE1 |  |  | 7918 | -0.170 | -0.3282 | Yes |
| 252 | ENC1 |  |  | 7921 | -0.170 | -0.3253 | Yes |
| 253 | GGCT |  |  | 7923 | -0.170 | -0.3222 | Yes |
| 254 | H2AFZ |  |  | 7924 | -0.171 | -0.3191 | Yes |
| 255 | TUBG1 |  |  | 7966 | -0.179 | -0.3206 | Yes |
| 256 | ANP32B |  |  | 7973 | -0.180 | -0.3180 | Yes |
| 257 | RAN |  |  | 7979 | -0.181 | -0.3152 | Yes |
| 258 | SNRPE |  |  | 7980 | -0.181 | -0.3119 | Yes |
| 259 | CHCHD3 |  |  | 7999 | -0.185 | -0.3106 | Yes |
| 260 | RPA3 |  |  | 8014 | -0.188 | -0.3088 | Yes |
| 261 | ST13 |  |  | 8037 | -0.191 | -0.3079 | Yes |
| 262 | HNRNPAB |  |  | 8043 | -0.192 | -0.3049 | Yes |
| 263 | HIST1H4C |  |  | 8075 | -0.198 | -0.3049 | Yes |
| 264 | ZFAND6 |  |  | 8115 | -0.207 | -0.3057 | Yes |
| 265 | NETO2 |  |  | 8118 | -0.208 | -0.3021 | Yes |
| 266 | SOX4 |  |  | 8123 | -0.209 | -0.2987 | Yes |
| 267 | RANBP1 |  |  | 8132 | -0.212 | -0.2957 | Yes |
| 268 | WDR83OS |  |  | 8146 | -0.214 | -0.2933 | Yes |
| 269 | MRPL17 |  |  | 8154 | -0.216 | -0.2902 | Yes |
| 270 | PMAIP1 |  |  | 8157 | -0.217 | -0.2864 | Yes |
| 271 | BTG1 |  |  | 8162 | -0.218 | -0.2828 | Yes |
| 272 | SET |  |  | 8175 | -0.221 | -0.2802 | Yes |
| 273 | UBE2I |  |  | 8202 | -0.229 | -0.2790 | Yes |
| 274 | MRPL18 |  |  | 8209 | -0.231 | -0.2755 | Yes |
| 275 | RHEB |  |  | 8224 | -0.234 | -0.2728 | Yes |
| 276 | PIN1 |  |  | 8225 | -0.234 | -0.2685 | Yes |
| 277 | CMC2 |  |  | 8236 | -0.236 | -0.2653 | Yes |
| 278 | HOXB2 |  |  | 8249 | -0.239 | -0.2623 | Yes |
| 279 | ATP6V1G1 |  |  | 8284 | -0.254 | -0.2616 | Yes |
| 280 | UPP1 |  |  | 8293 | -0.256 | -0.2578 | Yes |
| 281 | CALM2 |  |  | 8295 | -0.257 | -0.2532 | Yes |
| 282 | MAP3K7CL |  |  | 8298 | -0.258 | -0.2487 | Yes |
| 283 | EIF4EBP1 |  |  | 8316 | -0.262 | -0.2458 | Yes |
| 284 | PTTG1 |  |  | 8366 | -0.279 | -0.2465 | Yes |
| 285 | PFDN5 |  |  | 8373 | -0.282 | -0.2420 | Yes |
| 286 | DEK |  |  | 8376 | -0.284 | -0.2370 | Yes |
| 287 | YWHAH |  |  | 8384 | -0.289 | -0.2325 | Yes |
| 288 | CYCS |  |  | 8405 | -0.297 | -0.2294 | Yes |
| 289 | SNRNP25 |  |  | 8411 | -0.300 | -0.2244 | Yes |
| 290 | HMGN1 |  |  | 8432 | -0.308 | -0.2211 | Yes |
| 291 | POP7 |  |  | 8436 | -0.308 | -0.2157 | Yes |
| 292 | SNRPG |  |  | 8443 | -0.312 | -0.2107 | Yes |
| 293 | BEX1 |  |  | 8450 | -0.314 | -0.2056 | Yes |
| 294 | LYPD1 |  |  | 8460 | -0.321 | -0.2007 | Yes |
| 295 | C4orf3 |  |  | 8475 | -0.327 | -0.1963 | Yes |
| 296 | MLLT11 |  |  | 8489 | -0.335 | -0.1917 | Yes |
| 297 | H2AFV |  |  | 8497 | -0.340 | -0.1862 | Yes |
| 298 | TUBA1A |  |  | 8499 | -0.341 | -0.1801 | Yes |
| 299 | ID3 |  |  | 8502 | -0.341 | -0.1740 | Yes |
| 300 | SIVA1 |  |  | 8511 | -0.344 | -0.1686 | Yes |
| 301 | HMGA1 |  |  | 8519 | -0.346 | -0.1630 | Yes |
| 302 | PTMA |  |  | 8523 | -0.348 | -0.1569 | Yes |
| 303 | CHCHD2 |  |  | 8525 | -0.348 | -0.1506 | Yes |
| 304 | RPS27L |  |  | 8528 | -0.351 | -0.1444 | Yes |
| 305 | SAT1 |  |  | 8533 | -0.354 | -0.1383 | Yes |
| 306 | FABP7 |  |  | 8536 | -0.354 | -0.1320 | Yes |
| 307 | C12orf75 |  |  | 8548 | -0.363 | -0.1266 | Yes |
| 308 | S100B |  |  | 8562 | -0.373 | -0.1212 | Yes |
| 309 | PDCD5 |  |  | 8583 | -0.387 | -0.1165 | Yes |
| 310 | TMEM158 |  |  | 8586 | -0.391 | -0.1095 | Yes |
| 311 | LSM4 |  |  | 8611 | -0.413 | -0.1047 | Yes |
| 312 | SMS |  |  | 8624 | -0.430 | -0.0982 | Yes |
| 313 | RAD23A |  |  | 8628 | -0.433 | -0.0905 | Yes |
| 314 | HMGN2 |  |  | 8644 | -0.449 | -0.0840 | Yes |
| 315 | EEF1A1 |  |  | 8646 | -0.450 | -0.0758 | Yes |
| 316 | UBE2S |  |  | 8655 | -0.458 | -0.0683 | Yes |
| 317 | CDKN1A |  |  | 8657 | -0.461 | -0.0599 | Yes |
| 318 | PHLDA1 |  |  | 8670 | -0.476 | -0.0525 | Yes |
| 319 | RPL34 |  |  | 8671 | -0.476 | -0.0437 | Yes |
| 320 | COMMD6 |  |  | 8683 | -0.496 | -0.0358 | Yes |
| 321 | PRELID1 |  |  | 8684 | -0.498 | -0.0266 | Yes |
| 322 | TUBA1B |  |  | 8745 | -0.733 | -0.0202 | Yes |
| 323 | FTL |  |  | 8751 | -1.124 | 0.0000 | Yes |
Table: GSEA details [plain text format]

  

Fig 2: FELDMAN\_2020\_S-G2: Random ES distribution      
 Gene set null distribution of ES for **FELDMAN\_2020\_S-G2**
